# Developmental Assets of Adolescents and Young Adults With Chronic Illness and Comorbid Depression: Qualitative Study Using YouTube

**DOI:** 10.2196/23960

**Published:** 2021-02-16

**Authors:** Katherine Zheng, Maureen George, Eugene Roehlkepartain, John Santelli, Jean-Marie Bruzzese, Arlene Smaldone

**Affiliations:** 1 The Feinberg School of Medicine Center for Education in Health Sciences Northwestern University Chicago, IL United States; 2 Columbia University School of Nursing New York, NY United States; 3 Search Institute Minneapolis, MN United States; 4 Columbia University Mailman School of Public Health New York, NY United States

**Keywords:** adolescent development, chronic disease, depression, developmental assets, positive youth development, YouTube

## Abstract

**Background:**

Developmental assets provide a framework for optimizing development among adolescents but have not been studied in adolescents with chronic illness and comorbid depression, which is a group at risk for poor health outcomes. YouTube postings provide valuable insights to understand this understudied population.

**Objective:**

This study aims to explore asset development from the perspectives of adolescents and young adults (AYAs) with chronic illness and comorbid depression.

**Methods:**

YouTube was searched using 12 chronic illnesses (eg, diabetes) coupled with “depression” as keywords. Videos were included if they were uploaded by AYAs aged between 11 and 29 years and discussed living with chronic illness and depression during adolescence. Video transcripts were coded deductively for 40 internal and external assets that constitute the Developmental Assets Framework. Categories not captured by deductive coding were identified using conventional content analysis. Categories and their respective assets were labeled as being discussed either negatively or positively.

**Results:**

In total, 31 videos from 16 AYAs met the inclusion criteria. A total of 7 asset categories, support, constructive use of time, boundaries and expectations (external assets), identity, commitment to learning, positive values, and social competence (internal assets), reflecting 25 (13 internal; 12 external) assets, were discussed. Internal assets, particularly relating to identity, were commonly discussed by AYAs either in a negative way or fluctuated between positive and negative perspectives.

**Conclusions:**

In this sample of AYAs with chronic illness and comorbid depression, internal assets were commonly discussed in a negative way. Future research is needed to better understand how assets develop and if the Developmental Assets Framework adequately represents the experiences of this population.

## Introduction

### Background

The Positive Youth Development (PYD) theory has become a principal driver of national initiatives supporting the growth and development of youth [1], supporting a comprehensive approach to helping adolescents build on their strengths, rather than identifying and addressing their weaknesses. The Developmental Assets Framework provides a standard framework for conceptualizing the PYD theory, detailing a research-grounded set of 40 protective factors or developmental assets that build on one another to foster positive development [2]. In this framework, assets are categorized as either external, referring to the support and opportunities provided by a youth’s community (eg, family support), or internal, encompassing the commitments and values that youth build themselves through engaging in these opportunities and relationships (eg, academic motivation). Longitudinal studies have demonstrated that having a greater number of assets is associated with a number of positive academic, psychosocial, and behavioral outcomes, including a decreased risk of depression [3-6]. These outcomes have been well established among demographically diverse, healthy populations [2].

Up to 30% of adolescents live with a chronic illness [7], broadly defined as a long-term condition requiring ongoing care and management. Recent evidence suggests that developmental assets have a protective effect on mental health, including among adolescents with chronic illness [8-10], offering potential strategies to mitigate this population’s increased risk for depression [11,12]. However, as PYD is prevention oriented, applying a PYD lens to adolescents with chronic illness and comorbid depression is rarely undertaken. This represents a significant gap given that depressive symptoms can exacerbate chronic illness–related symptoms and worsen health outcomes [13,14]. An increased understanding of how adolescents with chronic illness and comorbid depression build assets is needed to inform care strategies to mitigate depressive symptoms in this population.

### Objectives

YouTube, a public video sharing site with more than 100 million active users, of which, 81% are aged between 15 and 25 years [15], can serve as a valuable secondary source of qualitative data [16-18]. Both registered and nonregistered users can post and consume an array of videos, including those discussing personal experiences. As adolescents with chronic illness and comorbid depression are often difficult to recruit for participation in research, YouTube may serve as an important avenue for gaining an understanding of their viewpoints. This qualitative study aims to explore asset development from the perspectives of adolescents and young adults (AYAs) who lived with chronic illness and comorbid depression during adolescence using videos from YouTube.

## Methods

### Design

This qualitative descriptive study used YouTube videos uploaded by AYAs with chronic illness and comorbid depression. The goal of qualitative description research is to provide rich accounts about a phenomenon that is not well understood, which can often serve to inform health care strategies by learning from those who experience the phenomenon under investigation [19]. This is accomplished by gathering the “surface of the data and events,” as described directly by individuals without a high degree of interpretation [20].

### YouTube Video Search and Selection

#### Eligibility Criteria

We included vlogs (ie, blog-style videos where individuals speak directly to the camera) if the individuals (1) were AYAs aged between 11 and 29 years who discussed experiences during adolescence; (2) indicated having a long-term, noncommunicable physical illness that requires ongoing self-management (ie, diabetes, cystic fibrosis, inflammatory bowel disease, rheumatoid arthritis, sickle cell disease, systemic lupus erythematous, Lyme disease, mast cell activation syndrome); (3) disclosed having current depression in either the title, description, or content of their video; and (4) shared experiences related to assets in the context of living with chronic illness and depression (eg, discussion on peer relationships). We excluded videos that (1) were not created by an AYA (eg, formal interviews, educational videos); (2) did not indicate age; and (3) were outside the scope of the research question. All videos uploaded since the inception of YouTube in 2005 were considered. One investigator (KZ) screened videos by title, thumbnail, and description and viewed any relevant videos in full to determine whether eligibility criteria were met. Video searches were conducted in June 2019 and repeated in September 2019 and January 2020 to ensure the identification of all relevant videos.

As YouTube users often upload multiple videos over time, we conducted a second search to identify supplementary videos from our selected participants to enrich our understanding of their experiences. Consistent with the process and criteria used to screen primary videos, we went to each AYA’s YouTube profile and screened these additional videos by title, thumbnail, and description to assess whether content could be relevant. Supplemental videos deemed relevant on the initial screen were then viewed in full and included if they met all eligibility criteria. Although any video from the same individual can add valuable insight into their lived experiences, we excluded supplemental videos that added no new and relevant information to the first video to keep our scope focused, given our inability to use a traditional interview guide.

#### Search Terms

We employed separate searches for each chronic illness that met the eligibility criteria. As the YouTube search engine is programmed to identify videos with exact or related search terms, we searched YouTube by pairing the names of each chronic illness of interest with the term *depression* (eg, *diabetes and depression*). We also conducted a broader search using the terms *chronic illness* and *depression* to identify videos that may have not been identified through the specific search terms listed above.

### Transcription

We downloaded the video transcripts directly from YouTube. We indicated the upload date and time of each video and merged multiple videos from the same individual into one transcript. One investigator (KZ) then watched all videos multiple times to clean transcripts and to create fieldnotes (descriptions of nonverbal behaviors and appearance). We extracted descriptive information on each AYA’s age, sex, chronic illness type, and whether the conversation reflected past or current experiences from each video. We also documented the number of views, likes, and dislikes the videos obtained, as these measures can be indicators of how popular a video is and whether viewers agree or disagree with the content being shared. We uploaded all transcripts to NVivo qualitative data analysis software to manage the qualitative data.

### Data Analysis

We analyzed transcripts using both directed and inductive content analysis [21]. For our deductive approach, we used a priori categories from the Developmental Assets Framework. This framework defines 8 broad categories, in which internal assets are categorized into individual strengths that reflect an adolescent’s (1) commitment to learning, (2) positive values, (3) social competence, and (4) positive identity, and external assets are categorized into environmental factors that provide youth with (5) support, (6) empowerment, (7) boundaries and expectations, and (8) constructive use of time. These 8 categories encompass 40 total individual assets that have been identified as fostering positive development among adolescents.

A total of 3 investigators (KZ, AS, and JB) independently coded the first 4 transcripts by labeling segments reflecting the 8 defined asset categories. We further coded passages to label the specific asset and characterized its discussion as positive or negative. These 3 investigators then met to reconcile the discrepancies and created an initial definition of the asset constructs. Using this initial template as a guide, one investigator (KZ) then independently coded the remaining transcripts, meeting weekly to review coding and obtain consensus. We iteratively updated this codebook throughout the coding process, compiling direct quotes from each transcript and grouping them by asset category and specific asset. We then created a template that allowed for side-by-side comparisons of assets used both positively and negatively in the discussion. This allowed for comparisons across transcripts and for the same AYA. Using this template, we then constructed a heat map to visualize the assets most commonly discussed and to identify where heavy fluctuations occurred between positive and negative discussions.

Through an iterative process, we also analyzed transcripts inductively to allow for the emergence of additional categories that did not fall under defined categories of the framework, but still provided context on youth development. Given that an interview guide was not used, we used the following a priori questions as guidance for this additional analysis, specifically assessing whether (1) AYAs discussed additional factors relating to the broad categories of the Developmental Assets Framework but not directly encompassed by the 40 specific assets listed; (2) whether AYAs mentioned additional types of activities, feelings, or behaviors that may impact their development; and (3) whether these new, emerging codes could potentially fall into the framework, and if not, what related context they arose from. We maintained an updated codebook of emerging codes and categories and their definitions, as determined by consensus. These categories were also marked to indicate whether the discussion was positive or negative. During weekly meetings, the 3 coders worked together to review inductive coding. Concurrent to the analysis of videos, we monitored for data saturation, the point at which no new information was learned. Typically, data saturation signals the end of data collection; however, with secondary analyses, only a finite number of data points (in this case, videos) are available without the option of returning to the field to continue data collection.

### Measures to Ensure the Trustworthiness of Qualitative Findings

We employed measures to enhance the trustworthiness of our findings [22]. To enhance confirmability (ie, the neutrality of interpretations) and dependability (ie, consistency of findings), we created a codebook, saturation table, and maintained a comprehensive audit trail by outlining each step of the decision-making process used for data collection, coding, and analysis. Bias was further reduced by multiple investigators with multidisciplinary expertise (ie, psychology and nursing), a form of investigator triangulation. Credibility was enhanced via weekly debriefings and immersion in the data, a form of prolonged engagement. To enhance transferability (ie, applicability of findings to like populations), we collected descriptive information about the YouTube community and videos.

### Ethics

The Institutional Review Board of Columbia University Medical Center designated this study as exempt from institutional review board review. We obtained a waiver of consent for the AYAs who uploaded the videos. Although YouTube is a public platform, we took measures to ensure the anonymity of the AYAs who uploaded videos by deidentifying all data. In addition, we searched quotes from each individual using Google and confirmed that the selected videos could not be identified by searching exact quotes that we extracted from the transcripts.

## Results

### Video Search Results and Descriptive Characteristics

**Figure 1:**
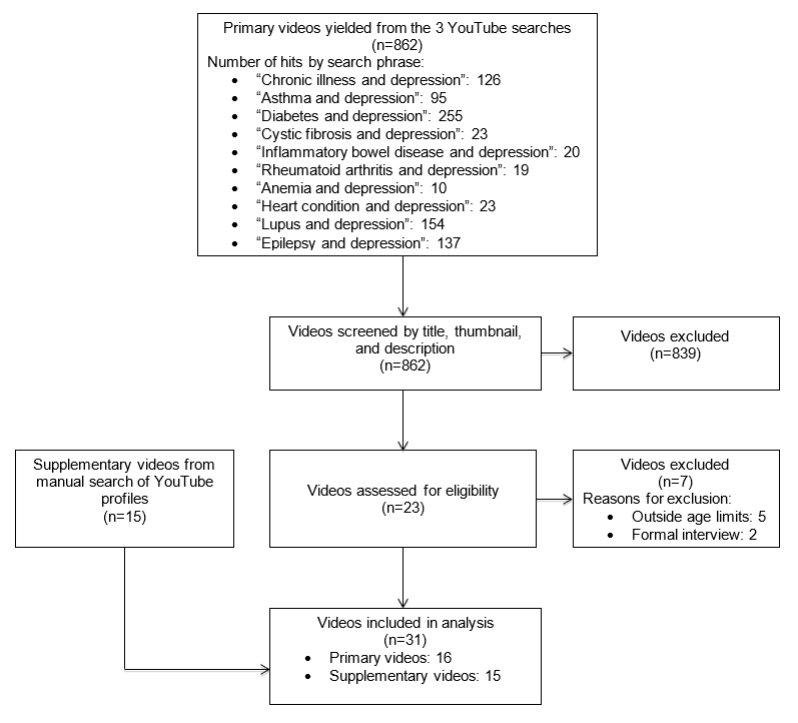
A flow diagram summarizing the YouTube video search selection.

Figure 1 depicts a flow diagram of the video selection process. Of the 862 primary videos screened, most were excluded for being (1) an educational health video, (2) uploaded by an individual outside the targeted age range, and (3) a clip from the news or a television show. Videos from 16 AYAs met all eligibility criteria (n=31 videos; range: 1-3 videos per person). A total of 15 primary videos were identified during the first search and one during the third search. Each of the 16 AYAs included in the initial search had uploaded anywhere from 1 to 528 total videos to their profiles. Of these supplementary videos, 15 (13 selected after the first search; 2 selected after the second search) met the eligibility criteria and added new information to the primary videos, resulting in 31 videos describing the experiences of 16 AYAs.

Table 1 summarizes the characteristics of the sample. AYAs were aged between 18 and 27 years during video recording. Most were female, reported type 1 diabetes, and had been diagnosed with their illness between the ages of 3 and 19 years. Collectively, the videos had obtained over 13,606 views, 1104 likes, and 12 dislikes. Overall, AYAs uploaded videos between November 5, 2013, and March 29, 2019, ranging in length from 3:13 min to 30:35 min. In total, 10 AYAs had more than 1 video included in our analysis. Videos from the same individual were mostly uploaded within the same month, with the exception of videos from 3 individuals that were uploaded across a 2- to 8-month time frame.

**Table 1:** Descriptive characteristics of the sample.

ID	Chronic illness	Sex	Age (years)^a^	Age of illness diagnosis (years)	Videos, n	Length of videos (mm:ss)	Total views, n	Total likes, n	Total dislikes, n
1	Type 1 diabetes	Male	22	19	1	8:57	972	29	1
2	Type 1 diabetes	Male	24	3	1	9:29	183	2	2
3	Type 1 diabetes	Female	22	15	1	13:22	2166	116	1
4	Type 1 diabetes	Female	20	12	1	8:26	152	1	0
5	Type 1 diabetes	Female	22	NR^b^	2	22:45	104	0	0
6	Type 1 diabetes	Male	21	18	3	28:00	617	54	0
7	Cystic fibrosis	Female	22	NR	2	20:49	482	5	4
8	Colitis	Male	22	10	2	25:56	415	2	0
9	Colitis	Female	27	17	2	16:26	353	11	0
10	Crohn disease	Female	22	14	1	30:35	312	6	0
11	RA^c^	Female	21	16	3	17:30	1309	77	1
12	SCD^d^	Female	22	NR	1	9:05	35	1	0
13	SLE^e^	Female	25	14	3	15:59	4772	609	2
14	MCAS^f^	Female	24	16	3	28:30	77	10	0
15	Lyme disease	Female	18	15	3	25:07	1087	38	0
16	IBS^g^	Female	22	19	2	26:59	570	53	1

^a^Age is documented as reported in the most recently uploaded video.

^b^NR: not reported.

^c^RA: rheumatoid arthritis.

^d^SCD: sickle cell disease.

^e^SLE: systemic lupus erythematosus.

^f^MCAS: mast cell activation syndrome.

^g^IBS: inflammatory bowel syndrome.

### Developmental Assets Identified by Deductive Coding

Table 2 provides definitions of all framework categories and their respective assets [2]; assets discussed in the videos are marked with an asterisk.

**Figure 2:**
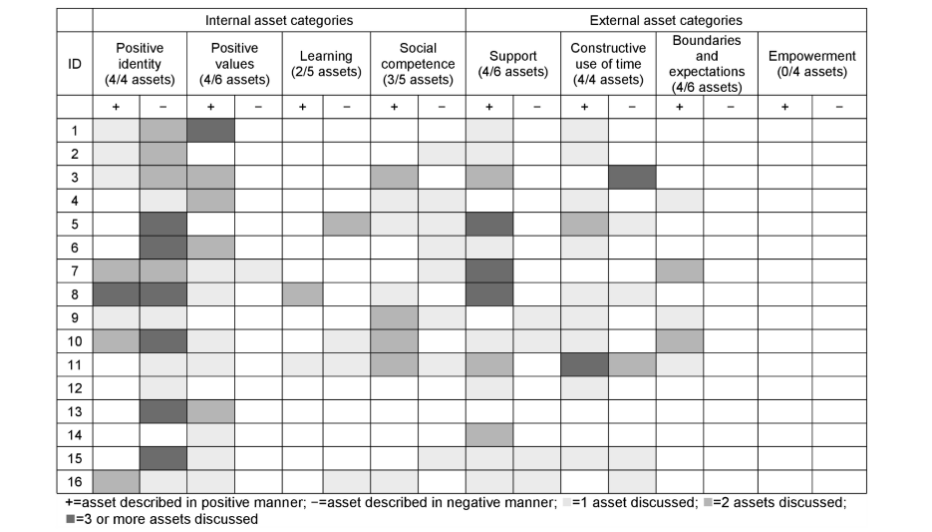
A heat map depicting the density of assets discussed within categories by each individual.

Figure 2 displays the heat map depicting the frequency and direction in which categories and assets are discussed. All but one category on the framework (empowerment) was discussed, reflecting 25 of the 40 assets.

**Table 2:** Developmental Assets Framework categories and asset definitionsa.

Category and definition				Asset and definition
**Internal assets**				
	**Positive identity**			
		Having high self-esteem, autonomy, holding life purpose, and optimism about the future	Personal power: feels he or she has control over “things that happen to me”^b^ Self-esteem: reports having a high self-esteem^b^ Positive view of personal future: optimistic about her or his personal future^b^ Sense of purpose: reports, “my life has a purpose”^b^	
	**Positive values**			
		Willingness to help others, be honest, and demonstrate respect for others, and give to the community	Caring: places high value on helping other people^b^ Honesty: tells the truth even when it is not easy^b^ Responsibility: accepts and takes personal responsibility^b^ Restraint: places importance on not being sexually active or using alcohol or drugs^b^ Equality and social justice: promotes equality and reducing hunger and poverty Integrity: acts on convictions and stands up for her or his beliefs	
	**Commitment to learning**			
		Appreciation of school and learning, reflected in ability to complete homework, be curious, and attend school	Achievement motivation: motivated to do well in school^b^ School Engagement: actively engaged in learning^b^ Homework: 1+ hours of homework done every school day Bonding to school: cares about her or his school Reading for pleasure: reads for pleasure 3+ hours per week	
	**Social competence**			
		Ability to express feelings, maintain peer relationships, say no to risky behaviors, and find positive ways to deal with hardships	Interpersonal competence: has empathy, sensitivity, and friendship skills^b^ Planning and decision making: knows how to plan ahead and make choices^b^ Resistance skills: can resist negative peer pressure and dangerous situations^b^ Cultural competence: comfortable with different cultural, racial, ethnic backgrounds Peaceful conflict resolution: seeks to resolve conflict nonviolently	
**External assets**				
	**Support**			
		Caring adult role models within an adolescent’s life, which may include parents, adults, teachers, and neighbors	Family support: received high levels of love and support from family life^b^ Other adult relationships: receives support from 3 or more nonparent adults^b^ Positive family communication: willing to seek advice and counsel from parents^b^ Caring school climate: school provides a caring, encouraging environment^b^ Caring neighborhood: experiences caring neighbors Parent involvement in schooling: parents actively help young person succeed in school	
	**Constructive use of time**			
		Extracurricular activities	Time at home: out with friends “with nothing special to do” 2 or fewer nights per week^b^ Youth programs: spends 3+ hours per week in sports, clubs, organizations in school, community^b^ Religious community: spends 1+ hour per week in activities in religious institution^b^ Creative activities: spends 3+ hours per week in lessons or practicing music, theater, other arts^b^	
	**Boundaries and expectations**			
		Expectations set at home, school, or in the neighborhood that teach adolescents responsible behavior	Positive peer influence: best friends model responsible behavior^b^ School boundaries: school provides clear rules and consequences^b^ Family boundaries: family has clear rules and monitors the young person’s whereabouts^b^ Adult role models: parents and other adults are modeling positive, responsible behavior^b^ Neighborhood boundaries: neighbors responsibly monitor young people’s behavior High expectations: both parents and teachers encourage the young person to do well	
	**Empowerment**			
		Climate at home or at school that allows youth to feel safe, valued, and appreciated	Community values youth: perceives that adults in the community value youth Youth as resources: given useful roles in the community Service to others: serves in the community 1+ hours per week Safety: feels safe at home, school, and in the neighborhood	

^a^The 40 Developmental Assets was used with permission from Search Institute. Copyright 1997, 2006 Search Institute, Minneapolis, MN.

^b^Asset reflected in YouTube videos.

#### Internal Assets

##### Positive Identity

All assets in this category were reflected across videos. Overall, the discussion about identity was more negative than positive. However, several AYAs described personal power and view of the future in both positive and negative ways. For example, one individual spoke about moments where his condition left him feeling powerless:

Diabetes for me, I thought that was the end of it. I thought I couldn’t touch sugar. I couldn’t touch any kind of drinks. I couldn’t eat much food. I couldn’t do anything...I thought, right, I’m effectively a broken toy now, so why would you want to be with me?

ID 1, male, age 22, type 1 diabetes

Later on, he expressed feeling more in control of his disease:

Knowing you can control your diabetes is giving me the strength, and it should give you the strength to say that if you can master this, and keep control of this, you can pretty much do anything.

Another individual spoke about previously viewing his future negatively due to his illness:

I went through a long two months of just...really just- just depression. My life was not going anywhere.

ID 8, male, age 22, colitis

However, he noted that more recently he had developed a more positive outlook:

Now I don't take anything for granted, you know, and everything that I receive, everything that goes well, I'm extremely grateful for.

Discussion on self-esteem was mostly negative, with several AYAs expressing struggles with self-confidence manifesting from visible side effects from their medication or symptoms:

My body wasn’t reacting very well to the insulin, so I started losing my hair, and as a teenage girl, hair and like body image, self-image is like really important...And when I looked into the mirror I saw someone who was sickly.

ID 3, female, age 22, type 1 diabetes

Several questioned their sense of purpose in living with an illness:

But the one thing that did stick with me was the question that I had during that time in my life, and it was ‘What’s the point of my life? What’s the point of living?’

ID 15, female, age 18, Lyme disease

##### Positive Values

All but two assets were reflected in the discussions. In contrast to the negative discussions around identity, almost all AYAs described the asset of caring in a positive way, encouraging their viewers to reach out to them for help:

If you need help, comment here. If you need advice, or anything...Anything that you want to talk to me about, just leave me an inbox message, or a personal message, or a comment below, and we’ll help you out for sure.

ID 4, female, age 20, type 1 diabetes

Except for one individual who admitted lying to hide self-harming behaviors from family, many pledged honesty in speaking to their viewers and the people around them:

I want to be transparent. I want you guys to truly see what I go through and not just, you know, my edited videos, but the ones that I just feel like saying what's on my mind.”

ID 13, female, age 25, systemic lupus erythematous

A few AYAs also discussed assets of restraint and responsibility, such as restraining from drugs and alcohol and responsibility for managing their illness:

I feel lucky and happy that I've been able to kind of kick depression right in the arse and say no, you're not taking over my life because the way I look at diabetes is it's not a disability, it's a responsibility.

ID 1, male, age 22, type 1 diabetes

##### Commitment to Learning

In total, 2 assets in this category, school engagement and achievement motivation, were discussed. A few individuals emphasized the importance of school and their future careers:

Um, you know, going to college, starting my career...I- I can't just take a year off, I can't do that. Um . . . especially in today's world with how the job market is, with how competitive everything is. I can’t do that.

ID 8, male, age 22, colitis

Conversely, others admitted that their conditions made them lose any desire to show up to class or complete school-related work:

And for the past few weeks, my depression has been pretty bad. I've not wanted to get out of bed. I’ve not wanted to do my college work.

ID 5, female, age 22, type 1 diabetes

##### Social Competence

AYAs discussed 3 assets within this category. Most AYAs positively described interpersonal competence through discussing their relationships with friends:

I would just talk to them, and I was friends with- my best friend has diabetes, had diabetes, and is still my best friend.

ID 4, female, age 20, type 1 diabetes

However, a few expressed struggling with interpersonal competence, such as one individual who had difficulty relating to others with diabetes:

I know a lot of people that have been diagnosed at a younger...uh at older ages like 10, 15, 11...And, I don't really know how to communicate with them about it, because so many of them, it's like, it's this life-changing thing. And to me, it's just...it's there because I've always dealt with it as long as I can really remember, you know? And, I mean yeah it sucks.

ID 2, male, age 24, type 1 diabetes

Several AYAs positively expressed their ability to plan and make decisions for their illness; however, one fluctuated between positive and negative discussions, voicing how she used to dwell on her depression:

But in asking all those questions about my depression and where is this coming from, I wasn’t actually looking into the future of actually making it better.

ID 9, female, age 27, colitis

Later, she explained how one positive decision to implement a better diet and care regimen had propelled her into a better lifestyle:

And that's the only reason I'm sitting here today, is because of that one little decision I made years ago.

A few AYAs also spoke about their inability to resist engaging in behaviors that they knew worsened their condition:

Because I'm binge-eating and- and like, I don't know how to deal with food. I never did. Like it’s- I don't know what to say right now.

ID 6, male, age 21, type 1 diabetes

#### External Assets

##### Support

Support was most commonly discussed across AYAs, with 4 of the 6 assets described. Almost all voiced the value of family support and other adult relationships, including health care providers:

Luckily, I've got a great doctor and a great support system behind me with my family and I feel like I can get through this and everything's gonna be okay.

ID 14, female, age 24, mast cell activation syndrome

Although support was mainly positive, some spoke negatively about their relationships with adults other than family, often due to skepticism from health care providers:

I was also dealing with not being believed by the medical community.

ID 15, female, age 18, Lyme disease

Another individual also described positive family communication, explaining how conversations with her father helped her deal with depression:

I came up to my room and my dad was lying on my bed and he asked me very specific questions about very specific things that I had been doing. And why, and what I was using, and what he could do to help.

ID 7, female, age 22, cystic fibrosis

##### Constructive Use of Time

All assets in this category were discussed, though not as frequently as other assets. Discussion around constructive use of time varied, with a few AYAs fluctuating between positive and negative discussions about time at home. One individual explained how when her condition worsened, it impacted her ability to engage in activities:

I didn't want to eat. I didn't want to hang out with friends. I didn't really want to talk. And I just really didn't want to socialize.

ID 11, female, age 21, rheumatoid arthritis

She also noted that when her symptoms lessened, she was able to enjoy activities again:

Once my RA symptoms actually started to lessen and the flares were way down, I started to do things that I enjoyed. I started to hang out with my friends...basically, just started to try enjoying my last year of college.

Others briefly touched upon the assets of youth programs, creative activities, and the religious community:

I know you can't see my wall, but I'm looking at it, and I have gotten into anime. It is phenomenal. I'm being more creative, I made a launcher for my room. I've got into art.

ID 5, female, age 22, type 1 diabetes

Conversely, a few lacked involvement in activities due to their illness:

I stopped doing things that made me happy, like writing for example. I was so into like creative writing, making stories, I had those fan fictions sometimes, just little things like that. And that was something I did to make myself happy. And also dancing, and just pretty much anything that I did before, I just stopped doing because I just didn’t feel like myself anymore.

ID 3, female, age 22, type 1 diabetes

##### Boundaries and Expectations

Although sparsely reflected, 4 assets within this category were discussed solely in positive light. For example, one individual discussed the asset, positive peer influence, by explaining how a friend to whom she reported her suicidal ideation had saved her:

Because, if you have that one person, they will be your saving grace, just like that one person was for me. And I'm so thankful for her. I'm so thankful she said something even though it was embarrassing and scary...I would not be where I am today without that stepping stone.

ID 7, female, age 22, cystic fibrosis

### Additional Categories Identified Through Inductive Coding

A total of 6 categories relevant to the broad categories of the framework but not encompassed by any of the specific asset definitions were also identified. We reached data saturation for all 6 categories early in the analysis, identifying no new categories after the second video. All categories were discussed by at least 3 individuals, with others (eg, learning about condition, sense of belonging, coping, caring online community) discussed by several.

#### Learning About Conditions

Several AYAs described their motivation to learn more about their illness and depression, including better ways to manage it:

I studied neuroscience to get a better handle on what was going on with my own body and also understand what was going on with, you know, the realm of chronic illness and mental illness and all of that stuff.

ID 14, female, age 24, mast cell activation syndrome

#### Sense of Belonging

Many discussed feelings of belonging to others who were in similar situations:

I found an amazing community of people who are like-minded, just like me, and that's...really incredible for me.

ID 10, female, age 22, Crohn’s disease

Conversely, others voiced feeling alone due to their illness:

And I feel lonely. Mentally, we can feel lonely in a room full of people, in a concert, anywhere. It's a mind state.

ID 6, male, age 21, type 1 diabetes

#### Coping

Almost all AYAs shared experiences of when they were able to cope positively or negatively with their illness and depression, with several fluctuations between the two. For example, one individual explained her previous battle with contemplating suicide as an escape from her illness:

Like, having chronic illness is...it ruins your whole life. And it makes you want to die. I mean, I can't tell you how many times in high school I contemplated suicide because I was so sick.

ID 10, female, age 24, mast cell activation syndrome

She later spoke about how she had found healthier ways to cope more recently:

And in college, I really just started getting a hold of myself, and my stress level, and my anxiety. So that helped a lot too.

#### Caring Online Community

Almost all AYA expressed receiving strong support from the online community, especially on YouTube:

RA [Rheumatoid Arthritis] community that I came across on Instagram and YouTube, and just being close with you guys have really helped, because I have been able to see how y'all handle RA and just actually have a group of people that I am close with and understand everything that I am going through.

ID 11, female, age 21, rheumatoid arthritis

#### Feeling Normal

One individual expressed coming to terms that her illness was a normal part of her life:

People can't tell unless you know my face like, you would think I'm normal. Like, I am normal, but God...um...It's draining. It's honestly draining. But I've come to it, like I've come to it in a light that it’s going to be normal, it’s going to be my life now.

ID 16, female, age 22, inflammatory bowel disease

Others voiced feeling *out of the norm* due to their illness:

I have suffered with depression, anxiety, uh...all sorts of minor issues...with sort of...myself, uh, feeling, you know, out of the norm.

ID 2, male, age 24, type 1 diabetes

#### Jobs

A few AYA also specified holding a job in their spare time:

Everything was going great! I was in a job, I was back on track, you know, I went to regular meetings, I felt on top of the world.

ID 1, male, age 22, type 1 diabetes

There were also a few individuals who disengaged from their job due to their illness:

Um, I went through another huge depressive episode when I- I lost my job...I was severely depressed.

## Discussion

### Principal Findings

Deductive findings provide important information as to the extent that adolescents with chronic illness and comorbid depression discuss developmental assets. Discussion of internal assets was more frequent than that of external assets. AYAs discussed assets from both a positive and negative perspective, demonstrating that asset development is not always a linear path forward. This was most evident for 2 internal asset categories (positive identity and social competence) and 1 external category (constructive use of time). Almost all AYAs in this sample discussed identity in a negative light, with some individuals fluctuating between positive and negative discussion, which often paralleled changes in reported illness and depression severity. It is important for professionals who work with adolescents to recognize that illness and depression may delay or dictate their ability to develop internal assets.

Contrary to identity, discussions regarding values were mostly positive. These AYAs emphasized helping viewers like them and pledged honesty about their experiences in their YouTube videos. Although this was a self-selected sample of AYAs who willingly uploaded videos on the web, this highlights the utility of using YouTube to identify individuals ready to discuss sensitive topics. As shown in 2 recent reviews, health education resources on YouTube have been examined quite extensively [23,24]; however, few studies have used this platform to explore experiences shared by those affected by health conditions [16,18,25]. As AYAs with chronic illness and comorbid depression are a difficult population to recruit, this study demonstrated the value of listening to their narratives on YouTube.

The Developmental Assets Framework builds on developmental theory of the bidirectional interaction of an adolescent’s internal capacity with their environment, suggesting that, at least in part, external assets (representing their environment) foster the development of internal assets [2]. Optimal development is most likely to occur when adolescents and their contexts are self-organized, with each reinforcing positive development of the other. In this sample, most individuals discussed their external assets, notably support, in a positive way; however, for the most part, this was not true for several internal assets. Most AYAs noted that their support systems helped them overcome difficult times, but their struggles with identity were ongoing, suggesting disequilibrium in their development that is likely associated with their illness and depression. In this case, their internal identity struggles may account for withdrawal from external assets, such as those in constructive use of time, with feelings of lack of power over their illness and many other areas of their lives. This may also explain the lack of reflection on empowerment opportunities in external assets.

### Implications for Clinical Practice

Monitoring assets relating to identity can be challenging given that they are expressed at the discretion of an adolescent. However, the narrative across AYAs revealed identifiable patterns of behavior that may serve as indicators of identity struggle. For example, individuals indicated withdrawing from activities that they were previously involved in, avoiding their friends, or disengaging from school, even if learning was important to them. These periods of withdrawal were expressed in conjunction with negative identity related to a depressive episode. As such, assets related to positive identity may need focused support (and opportunities to experience being empowered) when adolescents with chronic illness and comorbid depression experience depressive symptoms. Ongoing monitoring of not only an adolescent’s overall asset profile but also the types of assets exhibited in the context of the adolescent’s current condition and how they change can serve as an important avenue to provide higher quality of care for this population.

### Implications for Theory

The additional categories identified from the inductive analysis were more unique to adolescents with chronic illness. These findings may inform extensions of the Developmental Assets Framework tailored to the specific needs of this population while also aligning with broader PYD indicators. Another relationship to explore is the extent to which the current asset framework is associated with positive change in these potential assets related to understanding and coping with illness. An adolescent’s motivation to learn about their condition, sense of belonging, feeling normal, and coping may all be, at least in part, results of a strong base of other assets.

The fluctuation in discussions of assets between positive and negative is consistent with developmental theory. Human development is not a straight trajectory of positive growth toward adulthood, and development does not proceed from one step to the next and never *look back*. Rather, development is an iterative process in which persons interact with their contexts [1]. Some incidents may set them back, whereas other incidents may propel them forward. Thus, it is no surprise that many of these AYAs shifted their perspectives as they processed and integrated new experiences.

Related to internal assets, several individuals expressed a commitment to learning, but more in the context of learning about their respective health conditions and ways to manage them. In addition, having an illness appeared to be an additional factor that AYAs had to normalize and integrate into their identity. Consistent with our previous work [26], these additional categories reflect some of the common steps of understanding one’s illness, normalizing illness, overcoming limitations, and embracing responsibility that adolescents take in accepting their chronic illness. In addition, learning how to cope is a fundamental aspect of dealing with chronic illness during adolescence [27]. Much like discussions around assets within the framework, our findings illustrate that this sample fluctuated between positive and negative coping styles in relation to their depressive symptoms. Related to external assets, almost all AYAs expressed ways in which the YouTube community served as a strong form of support. Although it has been suggested that social media negatively impacts the mental health of adolescents [28], it is important to recognize the benefits that such platforms can offer. In addition, all of the videos we included obtained either no or very few dislikes and several videos had garnered tens to hundreds of likes, providing some indication that the content being shared was relatable or agreeable to the viewers on this platform. The Developmental Assets Framework does not specifically address the online community as a potential external resource for young people, a significant gap in the framework generally but particularly for this and other populations that find some of their most supportive communities on the web. Future research can focus on understanding developmental assets that are unique to the needs of those with chronic illness and comorbid depression.

### Limitations

Although this study sought to understand asset development among adolescents with chronic illness and comorbid depression, this sample may not be generalizable to non-YouTube users. It is plausible that those who were willing to share experiences may have presented a curated version of themselves that does not accurately depict their experience. In addition, the data were limited to what the individuals within the videos decided to discuss from their experiences. Despite the inclusion of multiple videos from the same individuals, these videos were mostly uploaded within the same month; therefore, we were unable to obtain a detailed sense of the temporal changes associated with asset development. Although 7 of the 8 categories on the Developmental Assets Framework were reflected, empowerment was not. It is possible that we could have heard discussion on the empowerment category if we had the option to sample more AYAs. Therefore, more research to explore asset development in this category is warranted. Relatedly, we were unable to gather information on the treatment, duration, or age at diagnosis of depression based on the content available. Furthermore, as individuals self-reported their diagnosis of chronic illness and depression, verification from either medical records or a health professional was not possible. As YouTube is used globally, there may also have been additional videos in other languages that were not included, limiting our ability to consider cultural generalizability. Finally, this qualitative study could not offer insight into whether and how the assets that were articulated by AYAs were related to positive outcomes such as coping with the illness, maintaining treatment protocols, and other well-being indicators.

### Conclusions

This study explored developmental assets from the perspectives of AYAs who lived with chronic illness and comorbid depression during adolescence using videos on YouTube. This sample discussed certain assets in both positive and negative ways. In addition, internal assets appeared to be more difficult to develop for these AYAs, despite external support from family, friends, and care providers. Additional categories highlight unique ways in which assets may apply to adolescents with chronic illness and comorbid depression (eg, learning about illness, support from health care providers). The findings of this study highlight the value of YouTube not only as a source of data but also as a support modality for AYAs who upload videos. More research is needed to understand how to better monitor and reinforce asset development among adolescents with chronic illness and comorbid depression.

## Abbreviations

AYA: adolescent and young adult
PYD: positive youth development
